# Supplementary material for: Bioactive-Loaded Hydrogels Based on Bacterial Nanocellulose, Chitosan, and Poloxamer for Rebalancing Vaginal Microbiota
Source: Pharmaceuticals (Basel). 2023 Nov 30;16(12):1671. doi: 10.3390/ph16121671 (PMC10748236; doi:10.3390/ph16121671)
Supplement: Supplementary file 1 [file pharmaceuticals-16-01671-s001.zip › pharmaceuticals-2709886-supplementary.pdf]

Supplementary materials

Bioactive-Loaded Hydrogels Based on Bacterial Nanocellulose, Chitosan, and Poloxamer for Rebalancing Vaginal Microbiota

Angela Moraru [1,2,†], Ștefan-Ovidiu Dima [3,†], Naomi Tritean [3,4,†], Elena-Iulia Oprița [5], Ana-Maria Prelipcean [5], Bogdan Trică [3], Anca Oancea [5], Ionuț Moraru [2], Diana Constantinescu-Aruxandei [3,*] and Florin Oancea [1,3,*]

[1] Faculty of Biotechnologies, University of Agronomic Sciences and Veterinary Medicine Bucharest, Bd. Mărăști Nr. 59, Sector 1, 011464 Bucharest, Romania; angela.moraru@pro-natura.ro

[2] S.C. Laboratoarele Medica Srl, Strada Frasinului Nr. 11, 075100 Otopeni, Romania; ionut.moraru@pro-natura.ro

[3] Polymers and Bioresources Departments, National Institute for Research and Development in Chemistry and Petrochemistry—ICECHIM, Splaiul Independentei Nr. 202, Sector 6, 060021 Bucharest, Romania; ovidiu.dima@icechim.ro (Ș.-O.D.); naomi.tritean@icechim.ro (N.T.); bogdan.trica@icechim.ro (B.T.)

[4] Faculty of Biology, University of Bucharest, Splaiul Independentei Nr. 91-95, Sector 5, 050095 Bucharest, Romania

[5] Department of Cellular and Molecular Biology, National Institute of Research and Development for Biological Sciences, Splaiul Independentei Nr. 296, Sector 6, 060031 Bucharest, Romania; iulia.oprita@incdsb.ro (E.-I.O.); anamaria.prelipcean@incdsb.ro (A.-M.P.); oancea.anca@gmail.com (A.O.)

* Correspondence: diana.constantinescu@icechim.ro (D.C.-A.); florin.oancea@icechim.ro (F.O.); Tel.: +40-21-315-3299 (F.O.)

† These authors contributed equally to this work.

Starting from the individual spectra of bacterial nanocellulose previously obtained and characterized [1], and of chitosan and Poloxamer 407, Table S1 with the characteristic absorption bands was generated. If we look at the individual FTIR spectra and at their molecular structures, we observe a number of different C-O bonds in all three (bio)polymers, with particular absorption bands. Understanding each particular vibration-rotation signal induced by the IR light energy absorption will allow further understanding of interaction mechanism and hydrogels' structural, muco-adhesive and delivery properties.

In Figure S1, the absorption bands of O-H in bacterial nanocellulose and chitosan appear in the region 3600-3300 cm^{-1}, while N-H in chitosan presents a wide absorption band between 3500-3000 cm^{-1}. Poloxamer (PX) has only two -OH groups at chain ends and is hydrophobic at room temperature, therefore it does not give a spectral signal in this region. But PX has more intense bands in the region 3000-2700 cm^{-1}, with a maximum at 2880 cm^{-1}, characteristic for the C-H bonds vibration in $-CH_2-$ polymeric chains of tri-block PEG-PPG-PEG and of particular methyl group $-CH_3$ in PPG with the specific absorption peak at 2970 cm^{-1}. The next diagnostic region, 2500-1700 cm^{-1}, is specific for triple and double bonds, without any absorption bands for the BNC-CS-PX systems.

The last diagnostic region (1700-1500 cm^{-1}) can be seen as the shrinking mirror of the 3400-3000 cm^{-1} region at half wavenumbers, also specific for O-H and N-H and for hydrogen bonds with water [2]. In this region also occur the signals of intra-molecular H-bonds vibration PyR-OH••O^{H}-PyR (where PyR refers to pyran ring) between neighbor glucopyranosic rings with absorption signal at 1645±5 cm^{-1} for dried BNC, respectively 1651±5 cm^{-1} for dried chitosan in Figure S1. Compared to BNC, chitosan has another band at 1582±10 cm^{-1} that can be assigned to intra-molecular H-bonds between -N$_{H2}$••HO- [3,4]. The band at ≈1650 cm^{-1} for chitosan is also known as the Amide I band of the remaining acetylated amino groups (-NH-$COCH_3$), with the main influence belonging to C=O group, and it is being used to determine quantitatively with a good precision the deacetylation degree (DD,%) of chitin, respectively the degree of acetylation (DA=100-DD, %) of chitosan using FTIR [5-8]. The absorption band at ≈1582 cm^{-1} is assigned to Amide II band in chitosan combined with Amine I, with the main influence assigned to -NH- group, and it is more susceptible to hydrogen bonding and peak variations in intensity and position, shifting from 1595 cm^{-1} at low degrees of N-acetylation to 1550 cm^{-1} at high %DA, which makes it unreliable for FTIR calculations of DA(DD), the best formula with simultaneous internal sample normalization being DA%=(A1650/A3450)x100/1.33 [5]. According to this formula, the DA for the chitosan used for our ternary hydrogels is 21.64%, respectively DD=78.36% (DD >75%, according to the supplier).

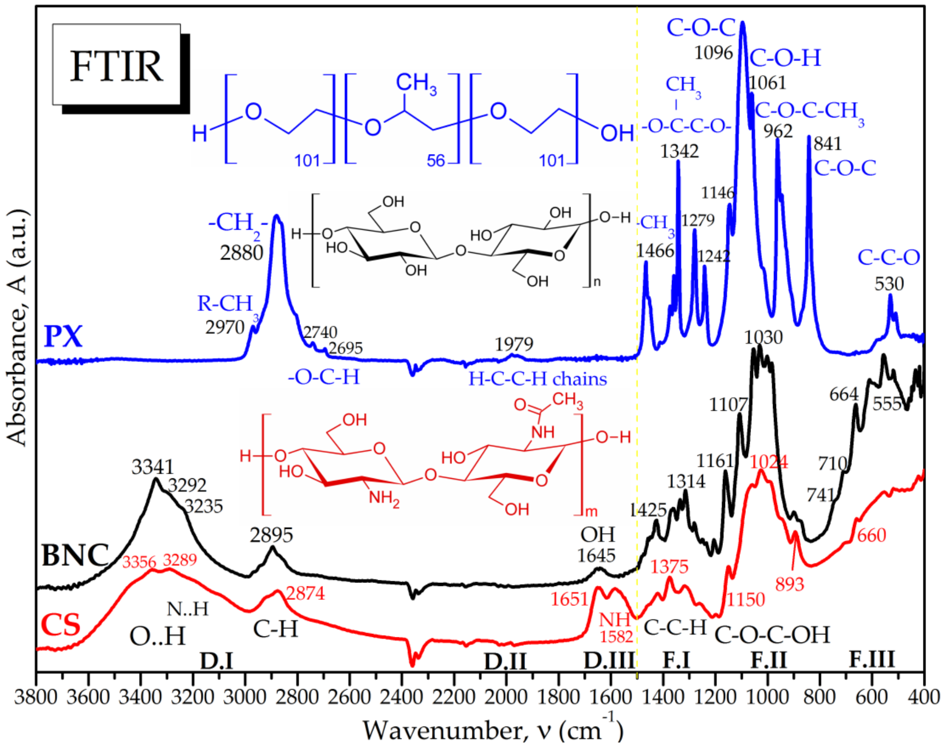

Figure S1. FTIR spectroscopy of ternary hydrogels components, respectively poloxamer (PX), bacterial nanocellulose (BNC) and chitosan (CS).

The fingerprint region 1500-400 cm^{-1} is more complex and rich in absorption bands because at lower energies particular vibration-rotations in-plane and out-of-plane occur for all atoms. This region can be further divided in 3 sub-regions with particular characteristics, named fingerprint F.I, F.II, F.III, in Figure S1. The fingerprint sub-region F.I (1500-1200 cm^{-1}) can be seen as the sub-region of macro-structures stretching vibrations, where the IR energy is still high and induces asymmetric and symmetric vibrations of large molecular structures, in particular for these samples the glucopyranosic rings in cellulose and chitosan, respectively the polymeric three-block chains in PX, together with their additional functional groups. Comparative spectra analysis between BNC and CS, respectively BNC-CS-PX, allows a better assignment of specific absorption bands based on similarities and differences. PX has the first absorption band at 1466 cm^{-1} with a shoulder at 1454 cm^{-1} assigned to C-H, mainly $-CH_3$ in PPG, respectively $-CH_2$-OH in PEG. For BNC/CS in F.I we can observe 9/6 weak-medium peaks, respectively 1456/1456, 1425/1420, 1369/1375, 1362/-, 1335/-, 1314/1317, 1281/-, 1248/1257 and 1206/1198 cm^{-1}, assigned as presented in Table S1, hopefully more relevant than in our first study on BNC [1]. Although the glucopyranosyl ring is similar in cellulose as in chitin/chitosan, there are some spectral differences induced by the functional groups, starting with the band at 1425 cm^{-1} having more shoulders and being more intense in BNC than CS due to more PyR-OH groups. The shoulder at 1456 cm^{-1} in both BNC and CS might belong to a C-OH group involved in hydrogen bonds, most probable -C3H-OH group which is usually involved in a strong axial hydrogen bridge with the heterocyclic O of the neighbor ring. At 1369 cm^{-1} for BNC, respectively at 1375 cm^{-1} for CS we assigned the asymmetric stretching of C-O in PyR-C6-OH, while BNC has an additional peak at 1362 cm^{-1} assigned to C2-OH, substituted in CS with the $-NH_2$ group. At 1206 cm^{-1} for BNC, respectively 1198 cm^{-1} for CS it appears a weak band, weaker for CS than BNC, which can be assigned to asymmetric stretching vibration of the glycosidic bond, while the symmetric stretching of PyR-O-PyR is assigned as the most intense band at 1024 cm^{-1} for CS, respectively 1030 cm^{-1} for BNC [4], as it will be argued in the F.II description.

In the F.I sub-region, PX has 3 types of C-O bonds with absorption bands at 1373, 1360 and 1342 cm^{-1}, the first two corresponding to PEG chains, respectively $-H_2$**C-O**H and HO-H_2**C-O-C**H_2- (-OH bending at 1361 cm^{-1} [9]), while the third and the strongest peak corresponding to C-O-C in PPG chain with the heaviest atomic system $-H_2$**C-O-C**H($-CH_3$)-CH_2-. Further in the F.I sub-region for PX are the bands at 1279 cm^{-1} assigned to $-H_2$C-O- deformation vibrations in PEG chain blocks, with a band at 1242 cm^{-1} assigned to >HC-O- deformation vibrations in PPG block.

The second fingerprint sub-region, F.II (1200-800 cm^{-1}), has the most intense absorption bands and is the most specific, since the medium IR energies here induce the deformation-rotation-vibration of all atomic bonds. This region is known as the polysaccharides band, characterized by C-O, O-C-O, C-OH, C-C and C-H deformation-rotation vibrations, while the sub-region 1200-1000 cm^{-1} [10] (1150-1060 cm^{-1} [4]) is particularly known as the region of ether bonds C-O-C, aliphatic or cyclic. The glycosidic bond PyR-O-PyR usually shows absorption bands for asymmetric stretching in the

region 1275-1200 cm^{-1} and for symmetric stretching between 1075-1020 cm^{-1} [4]. Starting with Poloxamer since has a simpler structure, we assigned the band at 1146 cm^{-1} to the vibration deformation of marginal -H_2C-OH, at 1096 cm^{-1} the deformation of -H_2**C-O-C**H_2- in lighter and longer PEG chains (C-O stretching at 1102 cm^{-1} [9]), -H_2**C-O-C**H(-CH_3)-CH_2- in heavier PPG chain at 1061 cm^{-1}, C-C-O-C in-plane vibration at 962 cm^{-1} with C-C shoulder at 945 cm^{-1}, ending with C-C deformation vibration in PEG polymeric chains at 841 cm^{-1}. In the same F.II sub-region, chitosan has the lowest number of well-defined bands, at 1024 cm^{-1} having the strongest signal that is usually assigned to the PyR-O-PyR glucopyranosic bond [11], flanked by 5 shoulders assigned to asymmetric and symmetric PyR-OH vibration at 1055 at 986 cm^{-1}, cyclic C-O-C in glucopyranosic rings at 1150 cm^{-1} most probable than glycosidic ring [12,13], respectively PyR-NH_2 at 941 cm^{-1} and aromatic C-C at 893 cm^{-1}. Cellulose has similar absorption bands, except the PyR-NH_2 band at 941 cm^{-1}, with additional mention that in cellulose the spectral bands are better defined due to weaker intra-molecular hydrogen bonds. A particular band for cellulose at 1107 cm^{-1} can be assigned to the C3-OH group substituted in chitosan with the -NH_2 group.

The last fingerprint sub-region, F.III (800-400 cm^{-1}), is the sub-region of simple bonds deformation vibrations in the presumed order of decreasing wavenumbers O-H, N-H, C-H, C-O, C-C and having the absorption bands assigned in Table S1, without particular aspects needed to be discussed.

Table S1. FTIR absorption bands of individual polymers BNC, CS and PX.

Bond/group	Wavenumber (ν), cm^{-1}			Observations / References (main tools [2-4])
	**BNC**	**CS**	**PX**	BNC-bacterial nanocellulose, CS-chitosan, PX-Poloxamer 407 (PEG-PPG-PEG), PyR-pyran ring
-H bonds with -OH	s3341±200	s3360±100	-	O-H bond (459 kJ/mol) is stronger than N-H (386 kJ/mol) and vibrates at a higher frequency; CS has two small peaks, around 3360 for -OH and at 3292 for -NH_2;
-H bonds with -NH_2	-	s3292±200	-	
H bridge with -OH	sh2970, sh2943	sh2924	sh2970	-asym. νCH_3 at 2962 cm^{-1}, -CH_3 in PPG; -H bridge with bound -OH, in correlation with the region at half ν=1485-1455 cm^{-1};
C-H in -CH_3, >CH_2	s2895±70	s2874±50	vs2880±90 sh2860	-C-H in BNC [1] and CS [14]; -CH_3 in PPG has the strongest signal at 2880 cm^{-1}, with a shoulder at 2860 cm^{-1} for -CH_2-;
O-H in -CH_2-OH	-	-	w2740	- bound -OH at chain ends;
H in C-H	-	-	w2695	- alone H in C-H bond to -CH_3 in PPG;
C-H, C-C	-	-	w1979±50	-particular for aliphatic chains in PX;
-OH in PyR-OH, PyR-CH_2-OH	s1638±60 m1645±50	-	-	- OH functional groups in cellulose involved in H-bonds with H_2O, as evidenced in Fig.FTIR3;
C=O in amide I and H-bonds with PyR-OH	-	s1651	-	Amide I [14,15]; -OH and H-bonds of N-acetyl group in partially deacetylated CS;
C-N-H in amide II	-	s1582	-	Amide II [14,15]; around 1595 at low DA, 1550 high DA & chitin; -H bonding increases the ν of amide II;
C-H coupled with >CH-OH, and correlated with H-bridge at 2xWN 2920-2970 cm^{-1}	sh1462 sh1456	sh1462 sh1456	s1466, sh1454	- δC-H in saccharides [2] with a shoulder for, most probably, -C6H_2-OH in Bnc and CS; -CH_3 in PPG gives the most specific band for PX at 1466 cm^{-1} with a shoulder at 1454 cm^{-1} for -CH_2-O-;

-CH$_2$-OH<br>PyR-OH	m1425	w1420	vw1412	-OH [15], more intense (m) in BNC than in CS (w) due to higher number of -OH, band highly susceptible for hydrogen bonding / hydration;<br>- (vw) in PX with only two -CH$_2$-OH at chain ends;
C-O in C-OH, Ar-C6-OH, O-C(=O)-CH$_3$	w1369	m1375	w1373	-C-O and C-H, weak in all samples, but best delimited in CS due to -CO-CH$_3$ in heavier acetyl moieties that increase asymmetry [14,15];
-OH in >CH-OH,<br>-CH$_2$-OH	w1362	-	m1360	- the vibration of >C2H-OH present in BNC but absent in CS, respectively -CH$_2$-O- in PX [9];
-CH$_2$-O-CH$_{(2)}$-	w1335	-	s1342	-PX has 3 types of C-O bonds (-H$_2$C-OH, -H$_2$C-O-CH$_2$- and -H$_2$C-O-CH(CH$_3$)-CH$_2$-), the heaviest group being the last one, giving the strongest peak;
C-N in Amide III PyR-NH-CO-CH$_3$<br>and PyR-O-PyR chain	-	w1317±30	-	- quite large band for C-N and C=O in Amide III band of partially acetylated chitosan moieties [14,15], possible coupled with glycosidic bond chain bending;
Hypothetically<br>PyR-O-PyR glycosidic linkage and chain effect	m1314	-	-	-band absent in mono- and oligosaccharides [2], or assigned for -CH$_2$- [16], or as above for CS, a hypothetically band for chain bending / oscillation at the glycosidic linkage PyR-O-PyR;
C-H in -CH$_2$-O-	w1281	-	s1279	C-H in PEG block chain;
-CH$_2$-O-CH$_{(2)}$-	w1248<br>w1234	w1257	s1242	-C-O- [15], most probable triple-bonded C in >CH-O-,<br>also >CH-O- in PPG block chain;
-CH$_2$-OH, -C6H$_2$-OH	w1206	w1198	-	-bending of -CH$_2$-OH (most probable -C6H$_2$-OH), weaker in CS than BNC due to stronger -OH..NH2-intramolecular H bonds, similar with trehalose, maltose and maltotriose [2];
PyR-O-PyR glycosidic link<br>C-O-C cyclic / aliphatic<br>PyR-C-OH / C-OH	m1161	m1150	s1146	-PyR-O-PyR asymmetric stretching of glycosidic linkage in polysaccharides [15,17]<br>- C-O-C cyclic in glucopyranosic rings, C-O-C aliphatic in PX, PEG chains [10]<br>- PyR-CH$_2$-OH in BNC and CS [14], respectively chain ends -CH$_2$-OH in PEG-PX;
PyR-OH / C-O	m1107	-	vs1096	C3-OH in BNC, respectively C-O-C in PEG-PX [9,10];
C-O-C aliphatic	-		s1061	-aliphatic C-O-C , C-O-C< in PPG-PX;
PyR-OH	s1055	w1059	-	C2-OH in BNC and CS [15];
PyR-O-PyR	s1030	s1024	-	-symmetric stretching of PyR-O-PyR glycosidic bond;<br>-C-O in CS [14,15];
C-OH / C-O	m1001	-	sh1015	-symmetric C3-O vibration in BNC ;
PyR-O-PyR / C-O-C/ C-C	m984	m986	s962 sh945	- glycosidic linkage in poly/oligo/saccharides [17], absent in monosaccharides and most affected by acidic hydrolysis of oligosaccharides [2], respectively C-O-C in-plane vibration with C-C shoulder in PX;
PyR-NH$_2$ / C-N	-	w941	-	C-N in PyR-NH$_2$ for chitosan;
PyR-O-PyR	w899	w893	-	- glycosidic link [15,18]
PyR-C / C-C	w872	-	vs841	- PyR-C in PyR-C-OH moiety for BNC has a higher liberty of vibration than the similar one in CS, while for PX vibrates C-C in polymeric chains;
O-H	w744,710	-	-	- in-plane scissoring and rocking vibrations of O-H in BNC;
O-H , N-H	-	w708	-	- in-plane scissoring and rocking vibrations of O-H and

				N-H in CS;
PyR C-H	m664	w660	-	- in-plane rocking vibrations of heterocyclic C-H;
C-H	w610	-	w584	- in-plane rocking vibrations of aliphatic C-H;
C-O	m555	w554	m530	- in-plane rocking vibrations of C-O;
C-C	<510	<510	<510	- in-plane rocking vibrations of C-C.

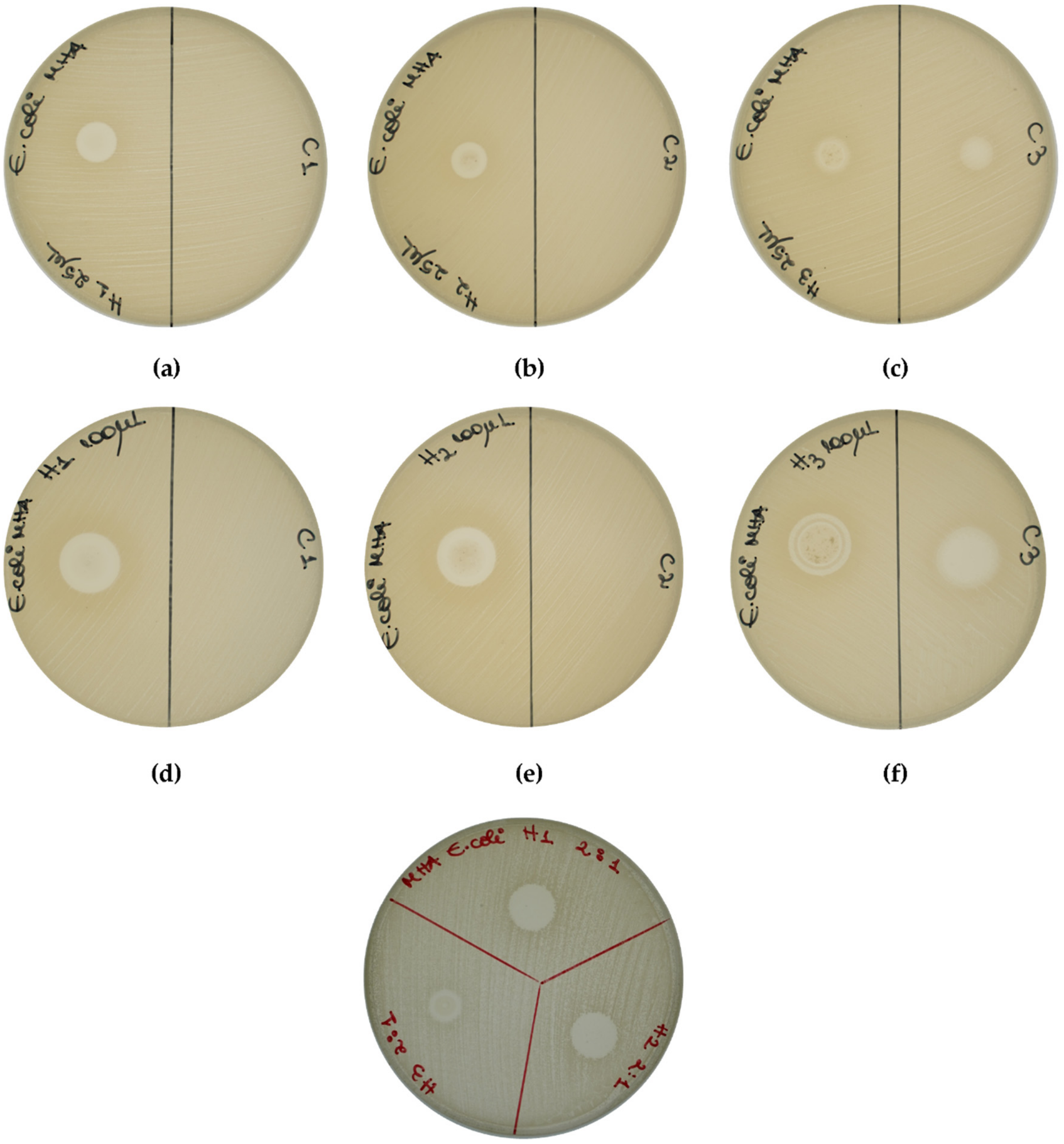

(a) (b) (c)

(d) (e) (f)

(g)

Figure S2. Qualitative screening of antibacterial activity: (**a**); Inhibition zone for *E. coli* treated with 25 µL H1, and C1; (**b**) Inhibition zone for *E. coli* treated with25 µL H2, and C2; (**c**) Inhibition zone for *E. coli* treated with 25 µLH3, and C3; (**d**) Inhibition zone for *E. coli* treated with 100 µL H1, and C1; (**e**) Inhibition zone for *E. coli* treated with 100 µL H2, and C2; (**f**)

Inhibition zone for *E. coli* treated with 100 μL H3, and C3;(**g**) Inhibition zone for *E. coli* treated with 25 μL 2:1 (v/v) diluted H1, H2, and H3; H1 – Hydrogel 1, H2 – Hydrogel 2, H3 – Hydrogel 3, C1 – Control of H1, C2 – Control of H2, C3 – Control of H3.

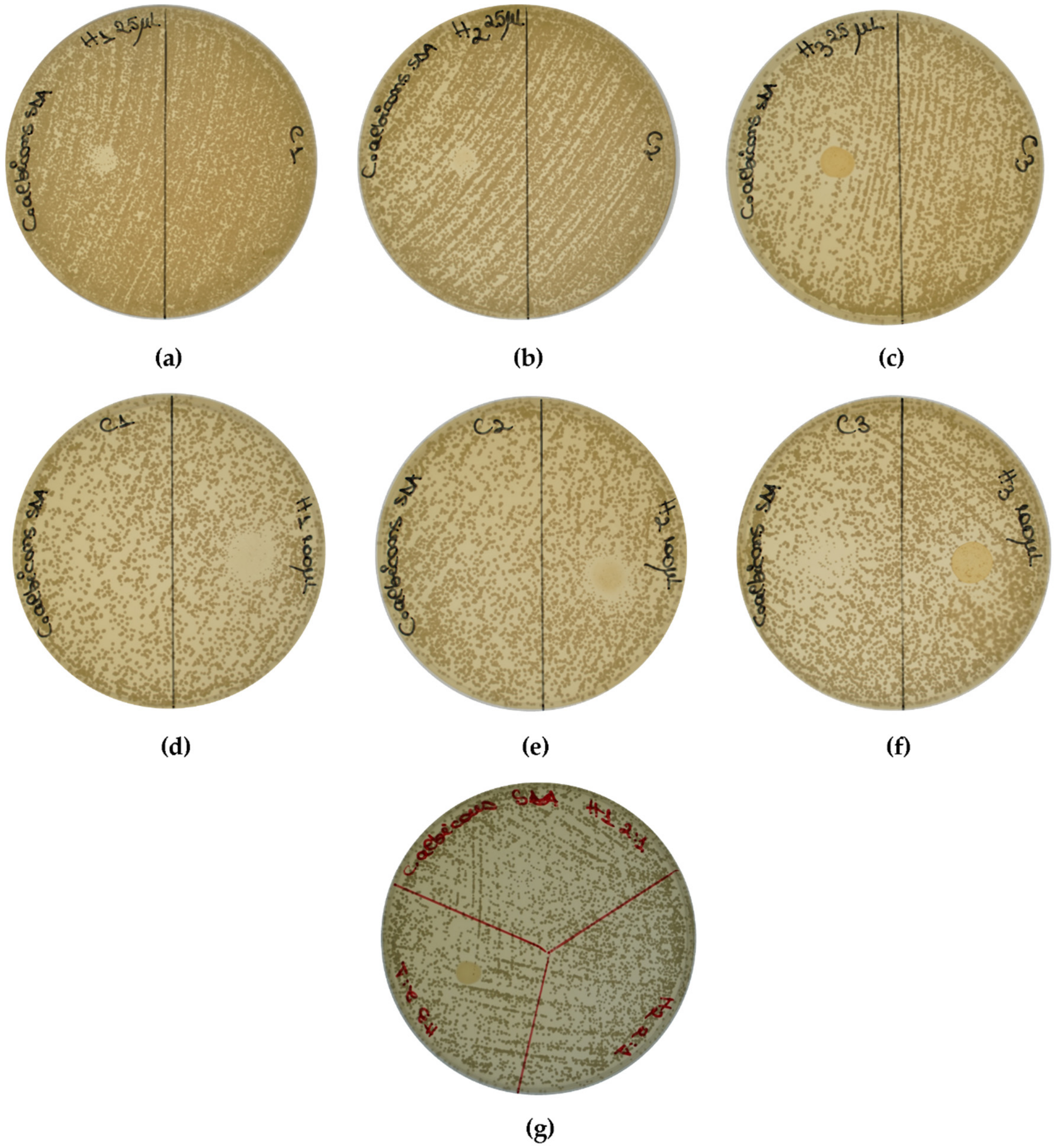

Figure S3. Qualitative screening of antimicrobial activity: (**a**); Inhibition zone for *C. albicans* treated with 25 μL H1, and C1; (**b**) Inhibition zone for *C. albicans* treated with 25 μL H2, and C2; (**c**) Inhibition zone for *C. albicans* treated with 25 μL H3, and C3; (**d**) Inhibition zone for *C. albicans* treated with 100 μL H1, and C1; (**e**) Inhibition zone for *C. albicans* treated with 100 μL H2, and C2; (**f**) Inhibition zone for *C. albicans* treated with 100 μL H3, and C3; (**g**) Inhibition

zone for *C. albicans* treated with 25 μL 2:1 (v/v) diluted H1, H2, and H3; H1 – Hydrogel 1, H2 – Hydrogel 2, H3 – Hydrogel 3, C1 – Control of H1, C2 – Control of H2, C3 – Control of H3.

Table S2. Rheological models and parameters for H1, H2 and H3 hydrogels, with and without mucin, in flow sweep mode.

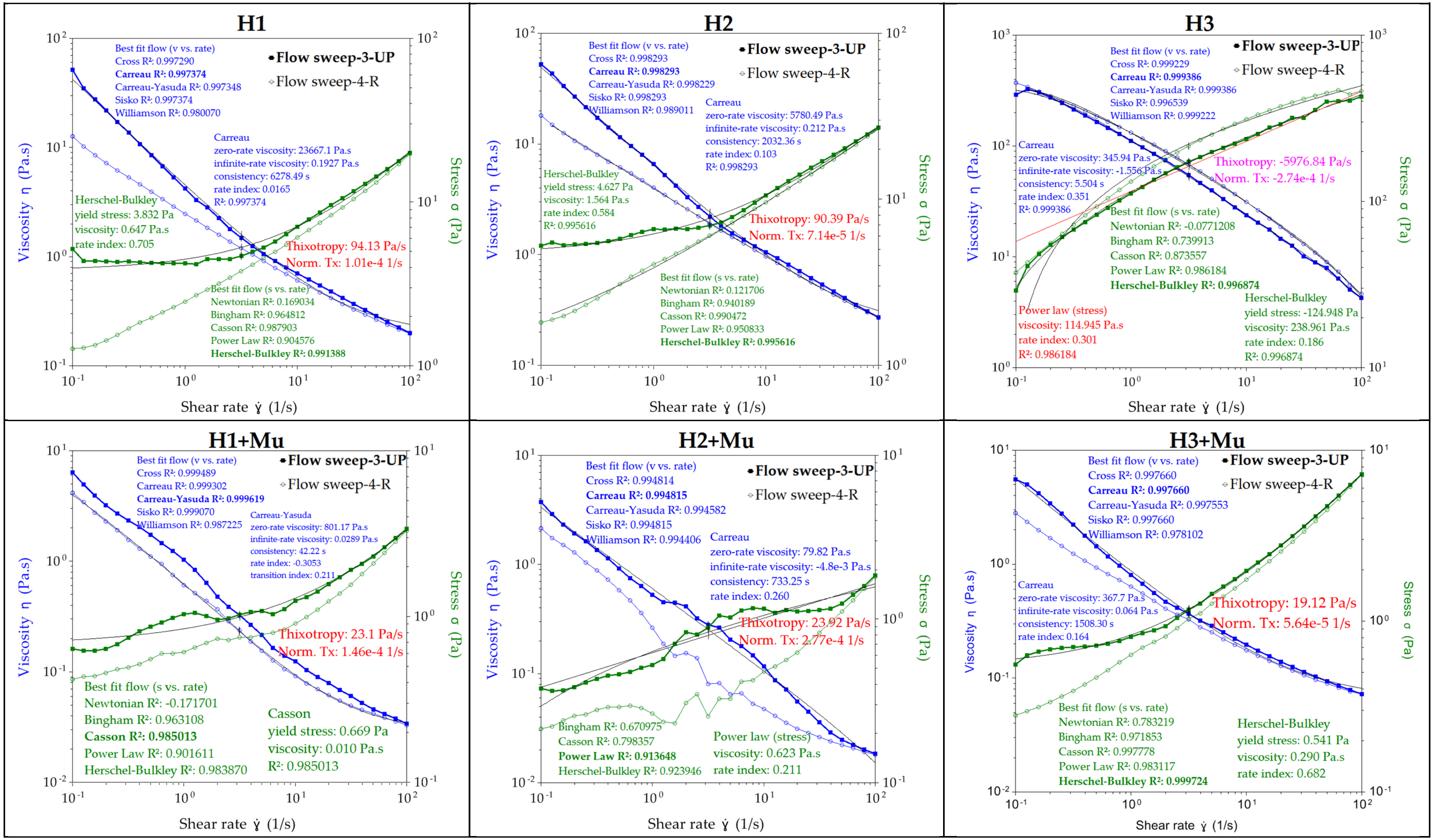

References

1. Dima, S.-O.; Panaitescu, D.-M.; Orban, C.; Ghiurea, M.; Doncea, S.-M.; Fierascu, R.C.; Nistor, C.L.; Alexandrescu, E.; Nicolae, C.-A.; Trica, B.; et al. Bacterial Nanocellulose from Side-Streams of Kombucha Beverages Production: Preparation and Physical-Chemical Properties. *Polymers* **2017**, *9*, doi:10.3390/polym9080374.
2. Kacurakova, M.; Mathlouthi, M. FTIR and laser-Raman spectra of oligosaccharides in water: Characterization of the glycosidic bond. *Carbohydrate Research* **1996**, *284*, 145-157.
3. Thomas, S.; Socrates, G. Spectroscopic tools; Infrared and Raman Characteristic Group frequencies. Available online: (accessed on June 2022).
4. Balaban, A.T.; Banciu, M.; Pogany, I.I. *Aplicatii ale metodelor fizice in chimica organica-RO (Applications of physical methods in organic chemistry)*; Editura Stiintifica si Enciclopedica: Bucuresti, 1983; p. 288.
5. Domszy, J.G.; Roberts, G.A.F. Evaluation of infrared spectroscopic techniques for analysing chitosan. *Die Makromolekulare Chemie* **1985**, *186*, 1671-1677, doi:https://doi.org/10.1002/macp.1985.021860815.
6. Baxter, A.; Dillon, M.; Taylor, K.D.; Roberts, G.A. Improved method for i.r. determination of the degree of N-acetylation of chitosan. *Int J Biol Macromol* **1992**, *14*, 166-169, doi:10.1016/s0141-8130(05)80007-8.
7. Khan, T.A.; Peh, K.K.; Ch'ng, H.S. Reporting degree of deacetylation values of chitosan: the influence of analytical methods. *J Pharm Pharm Sci* **2002**, *5*, 205-212.
8. Dash, M.; Chiellini, F.; Fernandez, E.G.; Piras, A.M.; Chiellini, E. Statistical approach to the spectroscopic determination of the deacetylation degree of chitins and chitosans. *Carbohydrate Polymers* **2011**, *86*, 65-71, doi:https://doi.org/10.1016/j.carbpol.2011.04.010.
9. Van Ngo, H.; Park, C.; Tran, T.T.D.; Nguyen, V.; Lee, B.J. Mechanistic understanding of salt-induced drug encapsulation in nanosuspension via acid-base neutralization as a nanonization platform technology to enhance dissolution rate of pH-dependent poorly water-soluble drugs. *European Journal of Pharmaceutics and Biopharmaceutics* **2020**, *154*, 8-17, doi:10.1016/j.ejpb.2020.07.001.
10. Cabana, A.; AitKadi, A.; Juhasz, J. Study of the gelation process of polyethylene oxide(a) polypropylene oxide(b) polyethylene oxide(a) copolymer (Poloxamer 407) aqueous solutions. *Journal of Colloid and Interface Science* **1997**, *190*, 307-312, doi:10.1006/jcis.1997.4880.
11. Abbasi, A.R.; Sohail, M.; Minhas, M.U.; Khaliq, T.; Kousar, M.; Khan, S.; Hussain, Z.; Munir, A. Bioinspired sodium alginate based thermosensitive hydrogel membranes for accelerated wound healing. *International Journal of Biological Macromolecules* **2020**, *155*, 751-765, doi:10.1016/j.ijbiomac.2020.03.248.
12. Fonseca-Garcia, A.; Caicedo, C.; Jimenez-Regalado, E.J.; Morales, G.; Aguirre-Loredo, R.Y. Effects of Poloxamer Content and Storage Time of Biodegradable Starch-Chitosan Films on Its Thermal, Structural, Mechanical, and Morphological Properties. *Polymers* **2021**, *13*, 10, doi:10.3390/polym13142341.
13. Bashir, M.; Syed, H.K.; Asghar, S.; Irfan, M.; Almalki, W.H.; Menshawi, S.A.; Khan, I.U.; Shah, P.A.; Khalid, I.; Ahmad, J.; et al. Effect of Hydrophilic Polymers on Complexation Efficiency of Cyclodextrins in Enhancing Solubility and Release of Diflunisal. *Polymers* **2020**, *12*, 17, doi:10.3390/polym12071564.

14. Costa, E.D.; Pereira, M.M.; Mansur, H.S. Properties and biocompatibility of chitosan films modified by blending with PVA and chemically crosslinked. *Journal of Materials Science-Materials in Medicine* **2009**, *20*, 553-561, doi:10.1007/s10856-008-3627-7.
15. Molina, R.; Jovancic, P.; Vilchez, S.; Tzanov, T.; Solans, C. In situ chitosan gelation initiated by atmospheric plasma treatment. *Carbohydrate Polymers* **2014**, *103*, 472-479, doi:10.1016/j.carbpol.2013.12.084.
16. Kacurakova, M.; Smith, A.C.; Gidley, M.J.; Wilson, R.H. Molecular interactions in bacterial cellulose composites studied by 1D FT-IR and dynamic 2D FT-IR spectroscopy. *Carbohydrate Research* **2002**, *337*, 1145-1153, doi:10.1016/s0008-6215(02)00102-7.
17. Nikonenko, N.A.; Buslov, D.K.; Sushko, N.I.; Zhbankov, R.G. Investigation of stretching vibrations of glycosidic linkages in disaccharides and polysaccarides with use of IR spectra deconvolution. *Biopolymers* **2000**, *57*, 257-262, doi:10.1002/1097-0282(2000)57:4<257::aid-bip7>3.0.co;2-3.
18. Kruer-Zerhusen, N.; Cantero-Tubilla, B.; Wilson, D.B. Characterization of cellulose crystallinity after enzymatic treatment using Fourier transform infrared spectroscopy (FTIR). *Cellulose* **2018**, *25*, 37-48, doi:10.1007/s10570-017-1542-0.
